# Acid-Sensing Ion Channels Expression, Identity and Role in the Excitability of the Cochlear Afferent Neurons

**DOI:** 10.3389/fncel.2015.00483

**Published:** 2015-12-22

**Authors:** Antonia González-Garrido, Rosario Vega, Francisco Mercado, Iván A. López, Enrique Soto

**Affiliations:** ^1^Instituto de Fisiología, Benemérita Universidad Autónoma de PueblaPuebla, Mexico; ^2^Dirección de Investigaciones en Neurociencias, Instituto Nacional de Psiquiatría Ramón de la Fuente MuñizMéxico D.F., Mexico; ^3^Department of Head and Neck Surgery, David Geffen School of Medicine, University of CaliforniaLos Angeles, CA, USA

**Keywords:** ASIC, inner ear, auditory, Corti, spiral ganglion, aminglycosides, acetylsalicylic acid, FMRFamide

## Abstract

Acid-sensing ion channels (ASICs) are activated by an increase in the extracellular proton concentration. There are four genes (ASIC1-4) that encode six subunits, and they are involved in diverse neuronal functions, such as mechanosensation, learning and memory, nociception, and modulation of retinal function. In this study, we characterize the ASIC currents of spiral ganglion neurons (SGNs). These ASIC currents are primarily carried by Na^+^, exhibit fast activation and desensitization, display a pH_50_ of 6.2 and are blocked by amiloride, indicating that these are ASIC currents. The ASIC currents were further characterized using several pharmacological tools. Gadolinium and acetylsalicylic acid reduced these currents, and FMRFamide, zinc (at high concentrations) and *N*,*N*,*N*’,*N*’–tetrakis-(2-piridilmetil)-ethylenediamine increased them, indicating that functional ASICs are composed of the subunits ASIC1, ASIC2, and ASIC3. Neomycin and streptomycin reduced the desensitization rate of the ASIC current in SGNs, indicating that ASICs may contribute to the ototoxic action of aminoglycosides. RT-PCR of the spiral ganglion revealed significant expression of all ASIC subunits. By immunohistochemistry the expression of the ASIC1a, ASIC2a, ASIC2b, and ASIC3 subunits was detected in SGNs. Although only a few SGNs exhibited action potential firing in response to an acidic stimulus, protons in the extracellular solution modulated SGN activity during sinusoidal stimulation. Our results show that protons modulate the excitability of SGNs via ASICs.

## Introduction

Acid-sensing ion channels belong to the ENaC*/*DEG family. Four *Asic* genes (Accn1-4) encode six different subunits: ASIC1a, ASIC1b, ASIC2a, ASIC2b, ASIC3 and ASIC4. These channels are expressed in the CNS and PNS. ASICs participate in synaptic plasticity, learning and memory, and fear conditioning (Wemmie et al., 2002, 2003; Du et al., 2014). In the retina, ASICs participate in synaptic transmission and neuroprotection. ASIC1a plays a significant role in cone function (Ettaiche et al., 2006), and ASIC2 plays a protective role against light-induced degeneration (Ettaiche et al., 2004). ASICs have been found to modulate the synaptic input of vestibular afferent neurons in the rat (Mercado et al., 2006, 2012) and axolotl (Vega et al., 2009).

ASICs participate in the pathophysiology of several CNS diseases. In epilepsy, ASIC1a activation in inhibitory neurons terminates seizure events (Ziemann et al., 2008). During ischemic episodes, blocking ASIC1a significantly reduces the infarct zone, protecting the brain from a major injury type. In the PNS, ASICs have been associated with inflammatory pain (Yagi et al., 2006; Deval et al., 2008). In a murine model of multiple sclerosis, ASIC1 gene deletion or the administration of specific ASIC blockers reduced the progression of neurodegeneration (Friese et al., 2007). Glutamatergic vesicles have a pH of approximately 5.7, and its release causes pH fluctuations in the synaptic cleft (Miesenböck et al., 1998; Du et al., 2014). The pH decrease in the synaptic cleft during synaptic activity is sufficient to activate ASIC, thus modulating postsynaptic membrane excitability (Du et al., 2014).

Glutamate is the primary afferent neurotransmitter in the auditory system. Its release from cochlear hair cells by ribbon synapses provides rapid and continuous input to afferent boutons, resulting in signals that strictly encode the time-course and intensity of sound (Goutman and Glowatzki, 2007). ASIC2 knockout mice exhibit increased resistance to noise-induced temporary threshold shifts (Peng et al., 2004), indicating a function of this subunit in hearing and the potentially harmful effects of acidosis (Sherwood et al., 2011). SGNs and the OC express ASIC3 (Hildebrand et al., 2004). Although ASIC3 knockout mice exhibit normal hearing, they develop hearing loss early in life (4 months of age) (Hildebrand et al., 2004). Additionally, the ASIC1b subunit was detected in SGNs and in the stereocilia bundle of mouse cochlear hair cells (Ugawa et al., 2006). Despite efforts to determine the role of ASIC channels in hearing, its role remains unclear. In this study, we characterize these ion channels in SGNs. Using the patch-clamp technique, we provide physiological and pharmacological evidence indicating the presence of all ASIC subunits in SGNs. RT-PCR and immunohistochemistry analyses revealed that four ASIC subunits are expressed in SGNs. Current-clamp experiments demonstrated that ASICs transmit excitatory input to SGNs, which may significantly modulate their electrical behavior.

## Materials and Methods

C57/BL mice of either sex were used for the experiments. The animal care and procedures were performed in accordance with the National Institutes of Health Guide for the Care and Use of Laboratory Animals and the *Reglamento de la Ley General de Salud en Materia de Investigación para la Salud* of the *Secretaría de Salud de México*. Protocols involving animal research were reviewed and approved by the Institutional Committee of Use and Care of Laboratory animals (CICUAL) from Research and Postgrade Vicerectory of the Benemérita Universidad Autónoma de Puebla (VIEP-BUAP). All efforts were made to minimize the suffering and to reduce the number of animals used, as outlined in the “Guide to the Care and Use of Laboratory Animals” issued by the National Academy of Sciences. Animals were supplied by the “Claude Bernard” animal facility of the Universidad Autónoma de Puebla where they were maintained in pathogen-free conditions using isolator for rats provided with disposable HEPA filters.

### SGN Cell Culture

Postnatal day 3 to 5 (P3–5) and 14 to 16 (P14–16) mice were used to obtain the SGN primary culture (Valdés-Baizabal et al., 2015). The mice were anesthetized using sevoflurane (Pisa Farmacéutica, Guadalajara, México) and were decapitated. SGNs were isolated and dissect from both inner ears and were incubated (30 min at 37°C) in 1.25 mg/ml trypsin and 1.25 mg/ml collagenase (both from Sigma–Aldrich, St. Louis, MO, USA)- diluted in L-15 medium (Sigma–Aldrich). After enzyme treatment, the ganglia were washed three times with sterile L-15 medium. The cells were mechanically dissociated using a Pasteur pipette and then seeded on 12 mm × 10 mm glass coverslips (Corning, Corning, NY, USA) pretreated with poly-D-lysine (Sigma–Aldrich). These neurons were incubated for 18–24 h in a humidified atmosphere (95% air and 5% CO_2_ at 37°C) using a CO_2_ water-jacketed incubator (Nuaire, Plymouth, MN, USA). The L-15 medium used for cell culture contained 15.7 mM NaHCO_3_ (Merck, Naucalpan, México), 10% fetal bovine serum (Gibco, Grand Island, NY, USA), 100 IU/ml penicillin (Lakeside, Toluca, México) and 15.8 mM HEPES (Sigma–Aldrich).

### Voltage- and Current-Clamp Recording

The patch clamp recordings were performed in SGN from P3–5 mice. Further, some recordings were performed in SGN from P14–16 mice were done to identify the ASIC current presence after the onset of hearing stage. To record ionic currents and voltage responses whole-cell patch-clamp technique was used. The cellular responses were examined according to standard voltage- and current-clamp protocols at room temperature (23–25°C) using an Axopatch 1D amplifier (Molecular Devices, Union City, CA, USA). The cells selected for recording were not adherent to other cells, did not display any neurite outgrowth, and exhibited a round, birefringent soma. Command-pulse generation and data sampling were controlled using pClamp 9.0 software (Molecular Devices) and a 16-bit data-acquisition system (Digidata 1320, Molecular Devices). The signals were low-pass filtered at 5 kHz and were digitized at 10 kHz. Patch pipettes were pulled from borosilicate glass capillaries (TW120-3; WPI, Sarasota, FL, USA) using a Flaming–Brown electrode puller (80/PC; Sutter Instruments, San Rafael, CA, USA). The electrodes typically displayed a resistance of 2–3 MΩ when filled with an internal solution of the following composition (mM): 10 NaCl, 125 KCl, 0.1 MgCl_2_, 10 EGTA, 1 Na-GTP, 2 Mg-ATP, and 10 HEPES (pH 7.3 adjusted with KOH). The osmolarity of the internal solution was adjusted to 300 mOsm. Approximately 80% of the series resistance was electronically compensated. Throughout the time-course of each experiment, the seal and the series resistance were continuously monitored to confirm stable recording conditions. The recording was not included in the analysis if the access resistance changed >10%.

For the current-clamp recordings, the cell membrane voltage was maintained near -60 mV. The cells were stimulated with sinusoidal current injection (33120A 15 MHz Arbitrary/Waveform Generator; Hewlett-Packard, Palo Alto, CA, USA). The frequency of the stimulus ranged from 10 to 30 Hz, and the stimulus amplitude ranged from 200 to 600 pA. As in the voltage-clamp recordings, the low pH solution was applied for 5 s.

### Solutions, Drugs, and Experimental Design

The cells were bath-perfused with extracellular solution containing (mM) 140 NaCl, 5.4 KCl, 2 MgCl_2_, 1.8 CaCl_2_, 10 glucose, and 10 HEPES. The pH of the extracellular solution was adjusted to 7.4 using NaOH. For those solutions whose pH was <6.5, the buffer MES was used instead of HEPES. The external solutions were adjusted to pH 8, 7.8, 7.6, 7.4, 7.2, 7.0, 6.5, 6.1, 5.5, 5.0, or 4.0. The osmolarity was monitored using a vapor pressure osmometer (Wescor, Logan, UT, USA) and was adjusted to 290 mOsm using dextrose. In some experiments, NaCl was equimolarly substituted with LiCl or Choline-Cl. A gravity-driven perfusion system maintained the external solution flow into the chamber at a rate of approximately 100 μl/min. To examine the responses of the cells to pH changes or to different drugs used, the recorded cells were perfused using a square-tube fast solution-changer (SF-77B Warner Inst., Hamden, CT, USA) change form one perfusion solution to another was in ∼20 ms. To examine the currents produced by the acidic solutions, the cells were voltage-clamped at a holding potential of -60 mV (approximately the normal resting potential of SGNs) and were perfused for 5 s with the test solution. In all of the experiments, at least two control responses were recorded before any experimental manipulation to assure that the cells maintain a stable acid-activated current. For perfusion of the drugs, the protocols were performed as described by Garza et al. (2010): (i) preapplication consisted of drug application for 10 s followed by perfusion of the pH 6.1 acid solution for 5 s; (ii) sustained application consisted of drug application for the preceding 10 s and during the 5 s acid solution perfusion; and (iii) coapplication consisted of drug application only during the 5 s acid solution perfusion.

The drugs used were: FMRFamide, ASA, amiloride, ST sulfate, Neo sulfate, and TPEN. Also, GdCl_3_ and ZnCl_2_ were used (all from Sigma–Aldrich). These drugs were freshly prepared before the experiments, and 10 μM capsazepine (Sigma–Aldrich) was added to all of the experiments to avoid the potential activation of TRPV1 channels in SGNs (Mercado et al., 2006).

### Data Analysis

For each experimental condition, a control and a washout recording were obtained. The data were analyzed oﬄine using Clampfit 9.2 software (Molecular Devices); the parameters measured in proton-gated currents were the *I*_peak_ amplitude (*I*_peak_) and the *I*_sus_ amplitude, measured as the mean of the current during the final 250 ms of the 5 s acid pulse. The current desensitization was fitted with a single exponential function obtaining a desensitization time constant (τ_des_). To construct the current versus pH curve, the *I*_peak_ values were normalized to that at pH 4. The pH-response curve was fitted with the function

$$Y=\min+\left(\max-\min\right)/\left(1+{\left(x/{EC}_{50}\right)}^{H}\right),$$

where *x* is the pH, max and min are the maximum and the minimum *I*_peak_, EC_50_ is the concentration at which 50% of the *I*_peak_ is detected, and *H* is the Hill slope constant. To evaluate the statistical significance of the data, a paired Student’s *t*-test or one-way ANOVA was used, and *P* < 0.05 was considered to be significant. The experimental data are presented as the mean ± standard error of the mean (SEM).

### Immunohistochemistry

C57/BL mice from postnatal day 25 (P25) were euthanized via an overdose of sevofluorane (Pisa Farmaceútica) and were perfused with 4% paraformaldehyde (pH 7.2) in 0.1 M sodium phosphate buffer. The temporal bones were removed from the skull, immersed in the same fixative for 4 h, and decalcified via immersion in a 5% EDTA phosphate-buffered solution for 5 days. The auditory bullae were further immersed in 30% sucrose. Midmodiolar sections (20 μm thick) were made using a cryostat (Microm HM 500, Zeiss, Oberkochen, Germany). The sections were mounted on glass slides (Superfrost-plus, Fisher Scientific, Leicestershire, England) and stored at -80°C until further use. For the study of ASIC expression in neurons after 24 h in culture the cells were fixed with 4% paraformaldehyde for 30 min and were washed in PBS.

For immunofluorescence, the tissue sections and cultured cells were incubated at room temperature for 30 min in a blocking solution containing 5% normal goat serum, 5% normal horse serum, and 0.5% BSA (fraction V, Sigma) in 0.1% Triton X100 in PBS. Next, tissue sections and cultured neurons were exposed to the primary polyclonal antibody against pan-ASIC1, pan-ASIC2, ASIC3, or ASIC4 diluted 1:200 (Abcam, Cambridge, MA, USA). To further corroborate ASIC expression in SGNs, a second experimental series (ADI series) of tissue sections were exposed to a different set of primary antibodies against ASIC1a, ASIC1b, ASIC2a, ASIC2b, ASIC3, or ASIC4 diluted 1:500 (ADI, San Antonio, TX, USA). All of these incubations were performed in a humidified chamber at 4°C for 48 h. After this incubation, the samples were washed three times for 10 min in PBS. Then, the samples were incubated in an Alexa Fluor 488-conjugated secondary antibody (1:1,000; Molecular Probes, Eugene, OR, USA) for 1 h at room temperature in the dark. The tissue sections were washed with PBS and then mounted using Vectashield solution (Vector Labs, Burlingame, CA, USA).

For immunoperoxidase staining, the sections were incubated for 1 h in a blocking solution containing 3% normal goat serum in 1% BSA (Sigma–Aldrich) and 0.5% Triton X-100 (Sigma–Aldrich) in PBS. Incubation in primary antibodies against ASIC subunits was done for 16 h at 4°C. The sections were washed with PBS (3 × 10 min) and incubated in biotinylated secondary antibody for 1 h (goat anti-rabbit IgG, 1:500) (Vector Labs), followed by incubation in Vectastain Elite ABC reagent (Vector Labs) for 1 h. Immunoperoxidase staining was performed using DAB reagent kit (Vector Labs). The slides were mounted using Permount mounting media (Fisher Scientific).

Negative controls were generated by incubating a pre-absorbed primary antibody in the corresponding blocking peptide (ADI) or by omitting the ASIC antibodies during the immunohistochemical procedure. The mouse DRG were used as positive controls for all subunits, and the cerebellar cortex was used as a positive control for ASIC2a. The tissue slices and cultured cells were observed using an Olympus FV1000 confocal microscope (Olympus, Tokyo, Japan). The ADI series of immunostained tissue sections was observed and imaged using an Eclipse E800 microscope (Nikon, Tokyo, Japan) equipped with an RTSlider spot digital camera (Diagnostics Instruments, Sterling Heights, MI, USA) and ImagePro Plus software (Media Cybernetics, Silver Spring, MD, USA). All histological figures were prepared using Adobe Photoshop software (Adobe Systems Incorporated, San Jose, CA, USA).

### RT-PCR

Spiral ganglion (SG) and Brain were dissected from P3-5 C57/BL mice and RNA was isolated using the TRIzol reagent method (Invitrogen). The RT-PCR experiments were done three times (*n* = 3); for each experiment, tissue from the inner ear of eight mice was pooled. The tissues were homogenized with a pellet pestle (Sigma–Aldrich) in TRIzol reagent, and total RNA was obtained according to the manufacturer’s instructions. The extracted RNA was treated with DNase I (Invitrogen) to eliminate the genomic DNA. The RNA quantity (>10 μg/μl) and purity (*A*_260_/*A*_280_ > 1.6) were verified by spectrophotometry, and the RNA integrity was confirmed via 2% agarose gel electrophoresis. cDNA was synthesized using the SuperScript First-Strand Synthesis Supermix kit (Invitrogen). PCR was done in a 25 μl reaction volume using cDNA as template. Primers to amplify the Asic1, Asic2, Asic3, and Asic4 mRNA were purchased from SABioscience-Qiagen, (Venlo, Netherlands; cat No. PPM32346A-200, PPM04118E-200, PPM38994A-200, PPM30788A-200, respectively). The 18S ribosomal primer (PPM57735E-200) was used as the constitutive-expression control. The predicted band size for each primer pair was 99, 169, 142, 116, and 129 for Asic1-4 and 18S, respectively. For PCR of negative controls, the RT procedure was omitted. Amplification was conducted using a Mini-Opticon thermal cycler (Bio-Rad, Hercules, CA, USA). The PCR products were labeled with ethidium bromide in a 2% agarose gel, and the image was taken in an UV transilluminator.

## Results

### ASIC Current Characteristics

**FIGURE 1:**
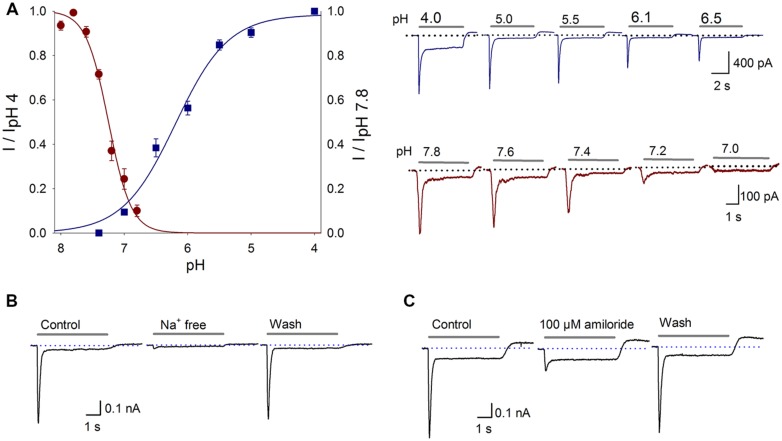
**Proton-gated currents in SGNs. (A)** Current–pH curves. ASIC activation (blue squares) was fitted with a sigmoidal equation, resulting in a pH_50_ of 6.17 ± 0.05 and a slope of 0.43 ± 0.05; *r^2^* = 0.99. The data represent the means ± SEM of at least six neurons. The top recordings show the activation of the proton-gated current by the different pH values indicated above each trace. The grey bar shows the pH change (5 s), and the dotted line indicates zero current. ASIC steady state desensitization (red circles) was fitted with a sigmoidal equation, resulting in a pH_50_ of 7.3 ± 0.03 and a slope of 0.14 ± 0.03; *r^2^* = 0.99. The points represent the means ± SEM of at least four neurons. The bottom recordings show the currents activated by the pH 6.1 solution after perfusion in the conditional pH. **(B)** Na^+^-free solution reversibly abolished the proton-gated current. **(C)** Amiloride (100 μM), a non-specific ASIC blocker, reversibly blocked the proton-gated current.

To determine the pH dependency of the proton-gated current of the SGN, the current was activated with extracellular solutions of different pH values: 4.0, 5.0, 5.5, 6.1, 6.5, or 7.0; the current-pH curve displayed a pH_50_ of 6.17 ± 0.05 and a slope of 0.43 ± 0.05 (**Figure 1A**, *n* ≥ 6). Therefore, in subsequent experiments, a pH 6.1 extracellular solution was used to activate the ASIC current. To further characterize the proton-gated current, the steady-state desensitization was determined. SGNs were perfused for 1 min with different pH extracellular solutions (8.0, 7.8, 7.6, 7.4, 7.2, 7.0, 6.8, or 6.6), and then current was activated using a pH 6.1 solution, the fitted current-pH curve displayed a pH_50_ of 7.3 ± 0.03 and a slope of 0.14 ± 0.03 (**Figure 1A**, *n* ≥ 4).

To determine if Na^+^ is the predominant permeant ion, a Na^+^-free extracellular solution was used (NaCl was equimolarly replaced with choline-Cl, pH 7.4 or 6.1). The *I*_peak_ of the proton gated current was reduced in 92% respect to control value with a Na^+^-free solution (*P* < 0.05, *n* = 10), indicating that the proton-gated current is essentially carried by Na^+^ influx (**Figure 1B**).

### Pharmacological Characterization of the ASIC Current

To further characterize this proton-gated current in SGNs, amiloride (a nonspecific ASIC blocker) was used. Amiloride (100 μM) reduced the acid-gated *I*_peak_ by 72 ± 2% (*P* < 0.05, *n* = 6) and increased the τ_des_ by 40 ± 9% (*P* < 0.05), although no significant change in the *I*_sus_ was detected (**Figure 1C**). The sensitivity of the proton-gated current in SGNs to amiloride and the finding that this current was attributed to Na^+^ indicate that it is an ASIC-mediated current.

**FIGURE 2:**
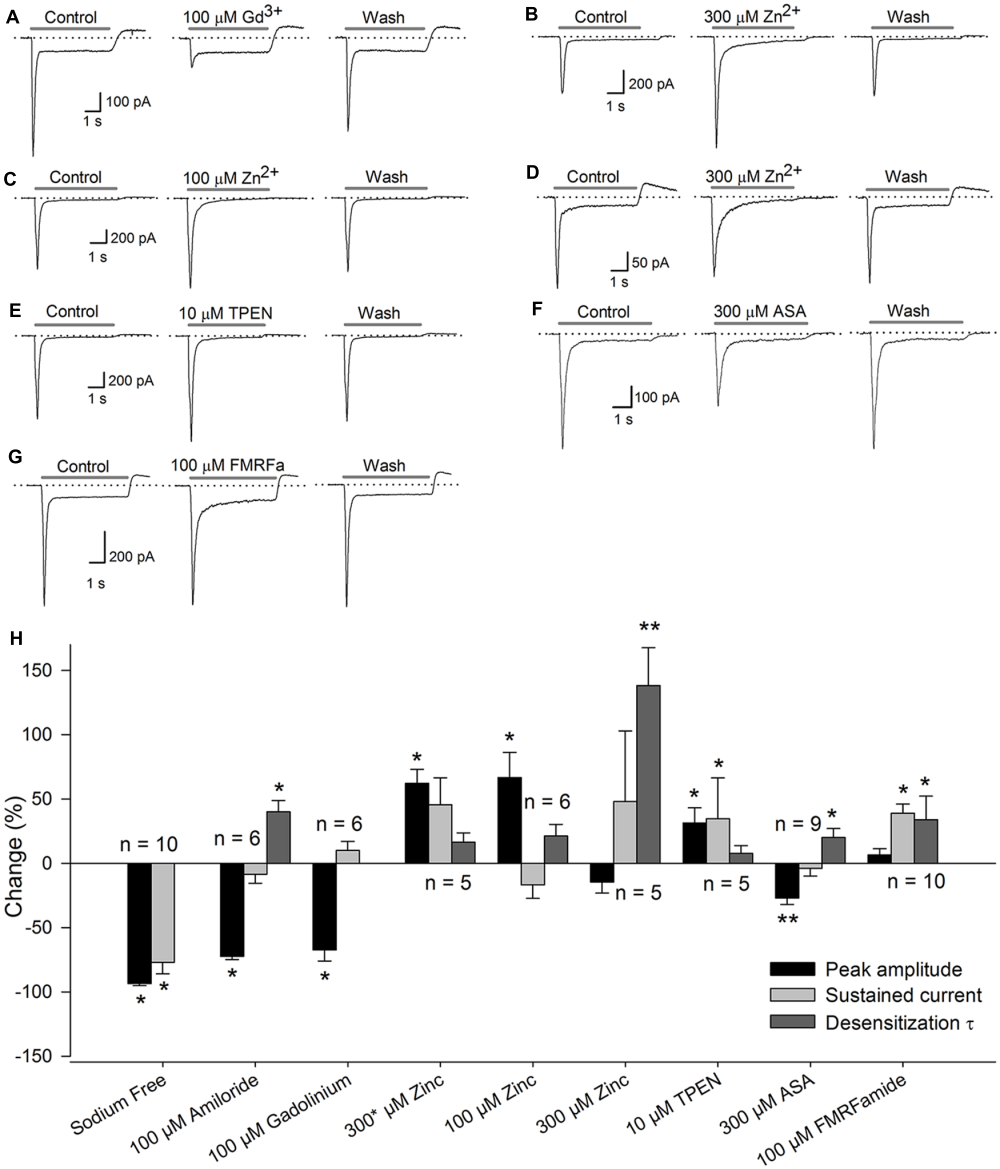
**Pharmacology of the ASICs in SGNs.** Recordings of the ASIC current activated by a pH 6.1 solution (gray bar) under control conditions, after drug application, or after drug washout. The dotted line represents zero current. **(A)** coapplication of 100 μM Gd^3+^ reduced the *I*_peak_. **(B)** Coapplication of 300 μM Zn^2+^ (^∗^) increased the *I*_peak_. **(C)** Sustained application of 100 μM Zn^2+^ also increased the *I*_peak_. **(D)** sustained application of 300 μM Zn^2+^ increased the τ_des_. **(E)** Coapplication of 10 μM TPEN increased the I_peak_. **(F)** Sustained application of 300 μM ASA significantly decreased the *I*_peak_ and increased the τ_des_. **(G)** Preapplication of 100 μM FMRFamide reversibly increased the τ_des_ and the *I*_sus_. **(H)** Bar graph summarizing the effects of the drugs used **(A–G)** The bars represent the means ± *SE* of the *I*_peak_ (black bars), the *I*_sus_ (light gray bars) or the τ_des_ (dark gray bars). The data are expressed as the percent-change relative to the control value. (^∗^*P* < 0.05, ^∗∗^*P* < 0.01, paired Student’s *t*-test).

Application of 100 μM Gd^3+^ reduced the *I*_peak_ by 67 ± 9% (*P* < 0.05, *n* = 6), although no change in the *I*_sus_ or the τ_des_ was detected (**Figure 2A**). It has been reported that high concentrations (high μM range) of Zn^2+^ increase the current in ASIC heteromers, including those containing the ASIC2a subunit (Baron et al., 2001), and that low concentrations (low nM range) of Zn^2+^ constitutively block the ASIC1a subunit (Chu et al., 2004). In our experiments, the coapplication of 300 μM Zn^2+^ to SGNs increased the current amplitude by 62 ± 11% (*P* < 0.05, *n* = 6), although no significant change in the *I*_sus_ or the τ_des_ was detected, indicating that the ASIC2a subunit is functionally expressed in the ASIC heteromers in SGNs (**Figure 2B**). By contrast, sustained perfusion of 100 μM Zn^2+^ increased the *I*_peak_ by 35 ± 11% (*P* < 0.05, *n* = 6) but did not affect the *I*_sus_ or the τ_des_ (**Figure 2C**). Additionally, sustained 300 μM Zn^2+^ application increased the τ_des_ by 138 ± 29% (*P* < 0.01, *n* = 5) but did not affect the *I*_peak_ or the *I*_sus_ (**Figure 2D**).

The sustained application of 10 μM TPEN (a high-affinity Zn^2+^ chelator) enhanced the ASIC current by 31 ± 12% (*P* < 0.05, *n* = 5) but did not alter the *I*_sus_ or the τ_des_ (**Figure 2E**), indicating that ASIC1a is functionally expressed in SGNs. The application of 300 μM ASA reduced the amplitude of the proton-gated current by 27 ± 5% (*P* < 0.01, *n* = 9) and increased the τ_des_ by 20 ± 7% (*P* < 0.05), but no significant change in the *I*_sus_ was detected (**Figure 2F**). Because ASA and Gd^3+^ modulate ASIC3 containing channels, these results indicate that ASIC3 is functionally expressed in SGNs.

FMRFamide-like peptides potentiate ASIC1- and ASIC3-containing channels (Askwith et al., 2000). In our experiments, pre-application of 100 μM FMRFamide increased the τ_des_ by 89 ± 22% (*P* < 0.05, *n* = 10) and the *I*_sus_ by 40 ± 12% (*P* < 0.05) but did not significantly affect the *I*_peak_ (**Figure 2G**).

The set of pharmacological tools used indicate that proton-gated currents in SGNs are mediated by ASIC channels composed of at least ASIC1, ASIC2, and ASIC3 (**Figure 2H**).

### Effect of Aminoglycosides on the ASIC Current

**FIGURE 3:**
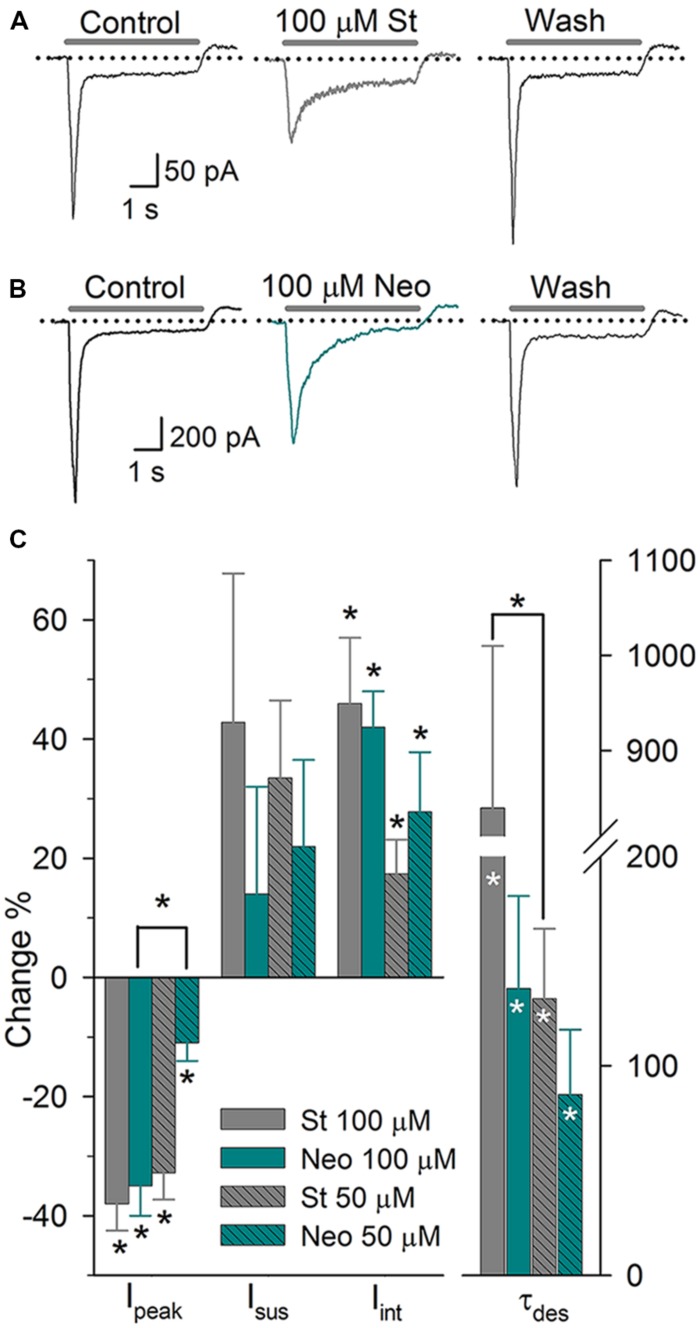
**The effect of aminoglycosides on the ASIC current in SGNs. (A)** Sustained application of 100 μM St produced a reversible reduction in the *I*_peak_ and the τ_des_. **(B)** Sustained application of 100 μM Neo produced similar effects but at a reduced potency. **(C)** Bar graph summarizing the actions of the aminoglycosides. The bars represent the means ± SE of the *I*_peak_, the *I*_sus_, the *I*_int_, or the τ_des_. The data are expressed as the percent-change relative to the control value (^∗^*P* < 0.05, paired Student’s *t*-test). Differences between concentrations were evaluated via one-way ANOVA (^∗^*P* < 0.05)

Aminoglycosides are widely used antibiotics that exert well-known ototoxic effects. Garza et al. (2010) found that in DRG neurons (DRGn), St, Neo, and gentamycin decrease the *I*_peak_, of ASIC current and increase its τ_des_ and the *I*_int_. In SGNs, 100 μM St (*n* = 10) and 100 μM Neo (*n* = 6) significantly decreased the peak of the ASIC current by 38 ± 5 and 26 ± 5%, respectively (*P* < 0.05 for both), and increased the τ_des_ by 840 ± 170 and 137 ± 44%, respectively (*P* < 0.05 for both) (**Figure 3**). The *I*_int_ was increased by 46 ± 11 and 42 ± 6% due to St and Neo application, respectively (*P* < 0.05 for both). Neither aminoglycoside altered the *I*_sus_. Applying 50 μM St (*n* = 7) decreased the *I*_peak_ by 33 ± 4% (*P* < 0.05), enhanced the *I*_sus_ by 34 ± 13% (*P* < 0.05), increased the *I*_int_ by 17 ± 6% (*P* < 0.05) and increased the τ_des_ by 132 ± 33% (*P* < 0.05). Applying 50 μM Neo (*n* = 6) decreased the *I*_peak_ by 11 ± 3% (*P* < 0.05), increased the τ_des_ by 107 ± 26% (*P* < 0.05), and increased the *I*_int_ by 28 ± 10% (*P* < 0.05) but did not significantly alter the *I*_sus_ (**Figure 3C**). The effects of St and Neo were dose-dependent (one-way ANOVA: *P* < 0.05). Increasing of τ_des_ causes an increase in the *I*_int_, consequently increasing Na^+^ entry, which most likely causes substantial depolarization and, therefore, hyperexcitation of SGNs, which may contribute to the ototoxic effects of aminoglycosides.

### ASIC Current in the Onset of Hearing

**FIGURE 4:**
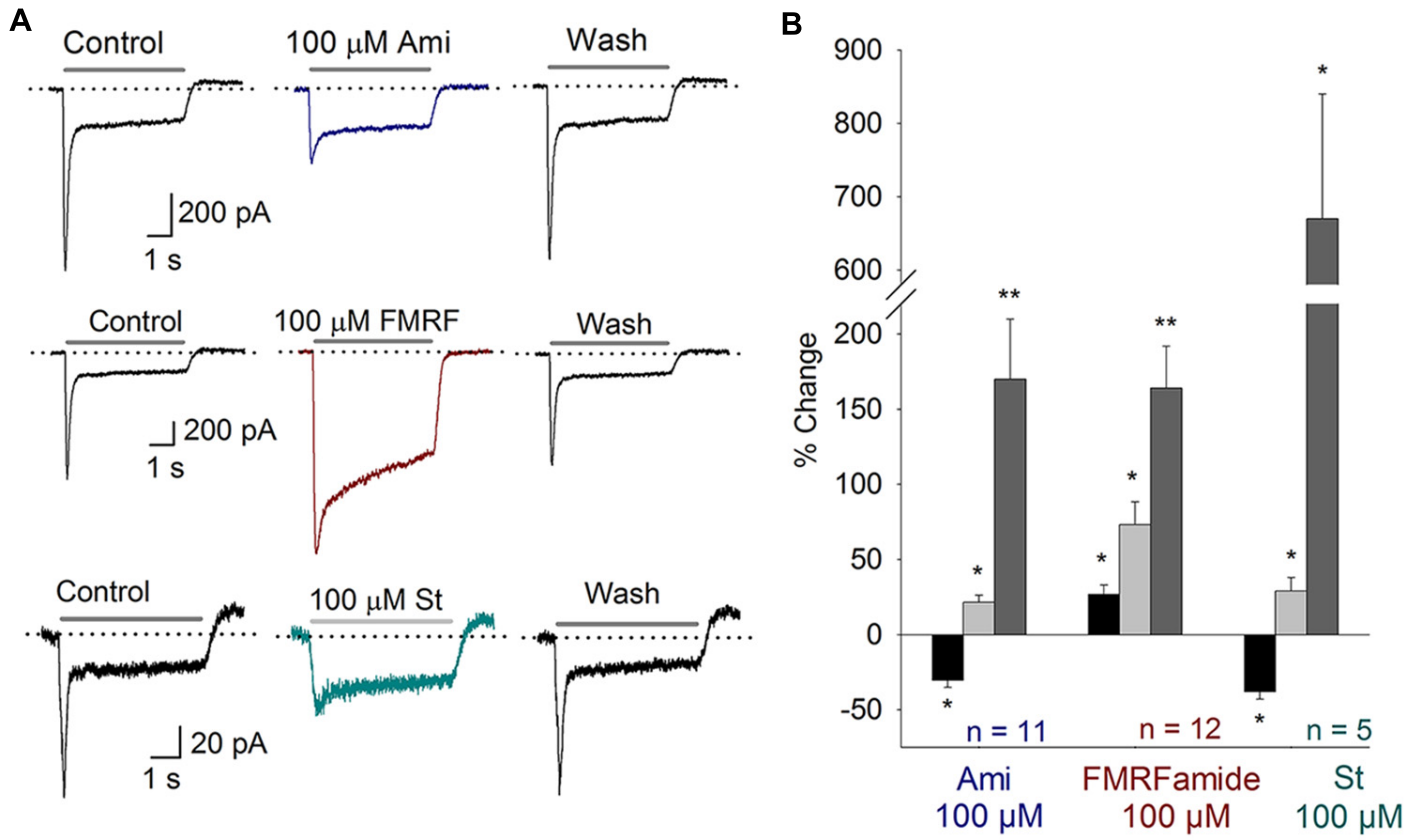
**ASIC currents in SGN from P14–16 mice. (A)** Top, Amiloride (Ami) blocks the peak of the ASIC current; Middle, FMRFamide (FMRF) increases the peak and sustained currents and reduces the desensitization rate; Bottom, St blocks the peak current, increases the sustained current and reduces the desensitization rate. **(B)** Bar plot summarizing the effects of the drugs from **(A)**. The bars represent the means ± SE of the *I*_peak_ (black bars), the *I*_sus_ (light gray bars) or the τ_des_ (dark gray bars). The data are expressed as the percent-change relative to the control value (^∗^*P* < 0.05, ^∗∗^*P* < 0.01, and paired Student’s *t*-test).

The onset of hearing is at P12 in mice. At this stage the IHC and afferent ends synapses are completely formed. Moreover, the activity of auditory nerve is mature between P12 and P20 (Shnerson and Pujol, 1981). In SGN cultured from P14–16 mice we also found electrophysiological and pharmacological evidence of functional ASIC currents. Amiloride (100 μM, *n = 11*) blocked *I*_peak_ 31 ± 5% (*P* < 0.05) and increased τ_des_ 170 ± 40% (*P* < 0.05) (**Figure 4A**). No effect on *I*_sus_ was observed. Second, FMRFamide (*n = 12*, 100 μM) increased the *I*_peak_ and *I*_sus_ as well as desensitization rate 27 ± 6, 73 ± 15, and 164 ± 28%, respectively (**Figure 4B**). And finally, 100 μM St (*n = 5*) blocked the *I*_peak_ 38 ± 5%, increased *I*_sus_ and desensitization rate 29 ± 9 and 670 ± 170%, respectively (**Figures 4A,B**). These results showed ASIC channels to be functionally present in mature cochlea.

Some effects on P14–16 mice were different from those observed in P3–5 mice. In the case of amiloride, both effects were significantly different from each other (*I*_peak_ and desensitization rate; ANOVA, *P < 0.01*). Besides, FMRFamide effects on the *I*_peak_ and *I*_sus_s were significantly different between these two stages (ANOVA, *P < 0.01*). All the effects observed in St treatment were not different between the two groups of mice.

### ASIC Subunit Expression

**FIGURE 5:**
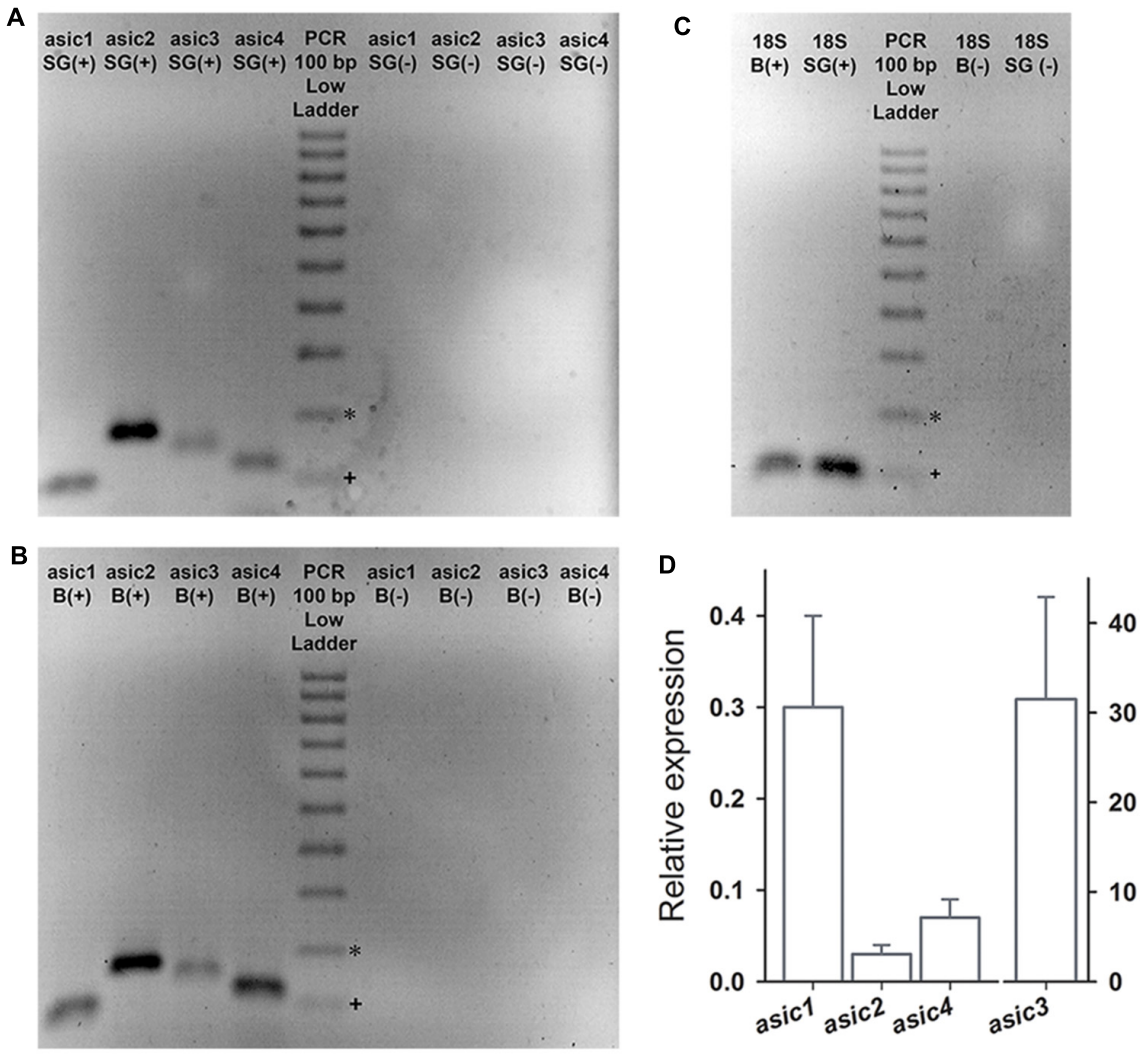
**The RT-PCR products were separated in a 2% agarose gel electrophoresis and were stained with ethidium bromide.** PCR was performed either in the presence (+) or absence (-) of reverse transcription (RT). **(A,B)** The PCR products corresponding to Asic1-4 were detected in the B and in the SG. **(C)** Expression of the housekeeping gene *18*S was detected in B and SG. **(D)** Bar graph showing the expression of Asic1–4 in SG relative to that in B. The expected band sizes (bp) were 99, 169, 142, 116, and 129 for *asic*1, *asic*2, *asic*3, *asic*4, and *18*S, respectively. ^∗^200 bp, ^+^100 bp.

RT-PCR was performed on the cDNA obtained from the SG to examine the ASIC subunits expression in the SGN. The brain (B) was used as positive control. The predicted sizes of the four *Asic* gene-related PCR fragments were detected in both the SG and the B (**Figures 5A,B**). mRNA corresponding to the 18S ribosomal subunit was used as constitutive expression control gene (**Figure 5C**). Relative to B *Asic* subunit expression levels were calculated using the 2^-ΔΔCt^ method (Livak and Schmittgen, 2001). The expression levels of Asic1, Asic2, and Asic4 were lower in the SG than in the B. By contrast, the Asic3 expression level was considerably higher, by approximately 30-fold, in the SG than in the B (**Figure 5D**). This result is in agreement with previous results comparing Asic3 expression between the brain and the cochlea (Hildebrand et al., 2004).

The high intensity of Asic2 band in SGNs suggests a high expression level; however, this implication is not conclusive because this method does not involve absolute quantification. The absence of genomic DNA from our RNA samples was confirmed based on negative controls in which reverse transcription was omitted (**Figure 5**). The expression of the four ASIC subunits demonstrated via RT-PCR is in agreement with the electrophysiological and pharmacological results.

### Immunohistochemical Localization of ASIC Channels in SG

The localization of ASIC subunit IR in the mouse cochlea was examined by immunofluorescence and immunoperoxidase staining.

**FIGURE 6:**
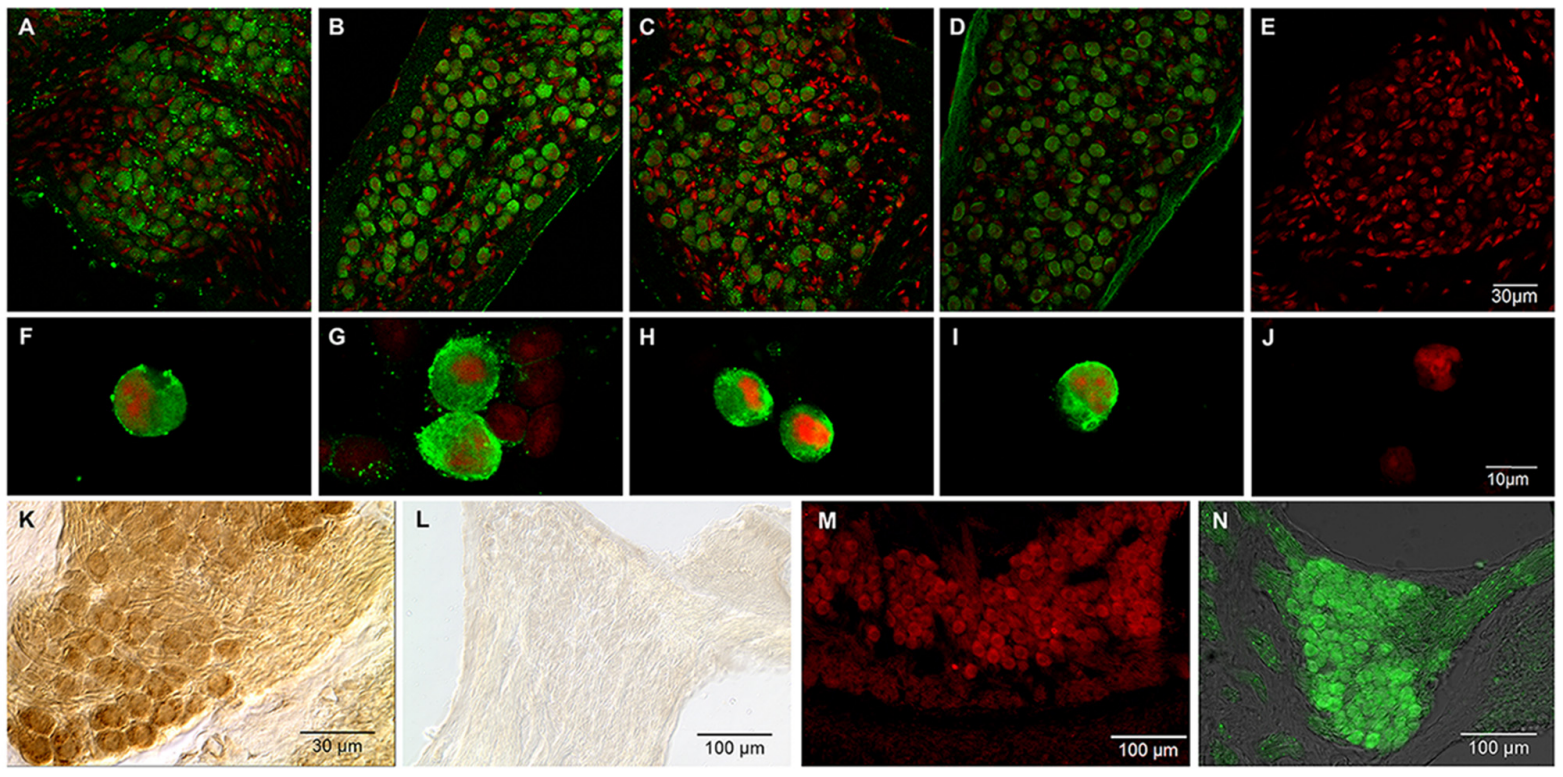
**Immunostaining for ASICs in SGNs.** Green, signal corresponding to a specific antibody for the indicated ASIC subunit; red, propidium iodide staining of the nuclei. **(A–D)** Immunohistochemistry of SG slices for ASIC subunits 1–4, respectively. **(F–I)** Immunocytochemistry of primary cultured cells for ASIC subunits 1–4 respectively. **(E, J)** control SG slices and cultured neurons, respectively, for which the primary antibody was omitted. In both the slices and the primary cultured neurons, all four ASIC subunits were detected. **(K,L)** Immunoperoxidase for ASIC1a and ASIC1b, respectively. Only ASIC1a subunit was localized to the SG. **(M)** Immunofluorescence staining for ASIC2a in the SG. **(N)** ASIC2b immunostaining in the SG.

Based on immunofluorescence, the four ASIC subunits (ASIC1, 2, 3, and 4) were detected in SG (**Figures 6A–E**). ASIC3 and ASIC2 displayed the highest level of staining in SGNs. All of the ASICs displayed uniform staining of the SGNs. Immunostaining of primary cultured neurons (**Figures 6F–J**) demonstrated the presence of the ASIC1, 2, 3, and 4 subunits; the corresponding antibodies uniformly stained the cell bodies.

An additional experiment (ADI series) using antibodies from a different source was performed to confirm the expression of ASIC subunits in SG and to determine the IR of the ASIC1 and ASIC2 splice variants (ASIC1a, ASIC1b, ASIC2a, and ASIC2b). ASIC2a and ASIC2b were detected in mouse SGNs via immunofluorescence (**Figures 6K–N**). This result is in agreement with previous results in which ASIC2a and 3 were detected in the mouse cochlea (Hildebrand et al., 2004; Peng et al., 2004).

The IR of ASIC1a and ASIC1b were examined using immunoperoxidase staining. The ASIC1a subunit was detected in the SG. Interestingly, ASIC1b was not detected in the SG (**Figures 6K,L**).

**FIGURE 7:**
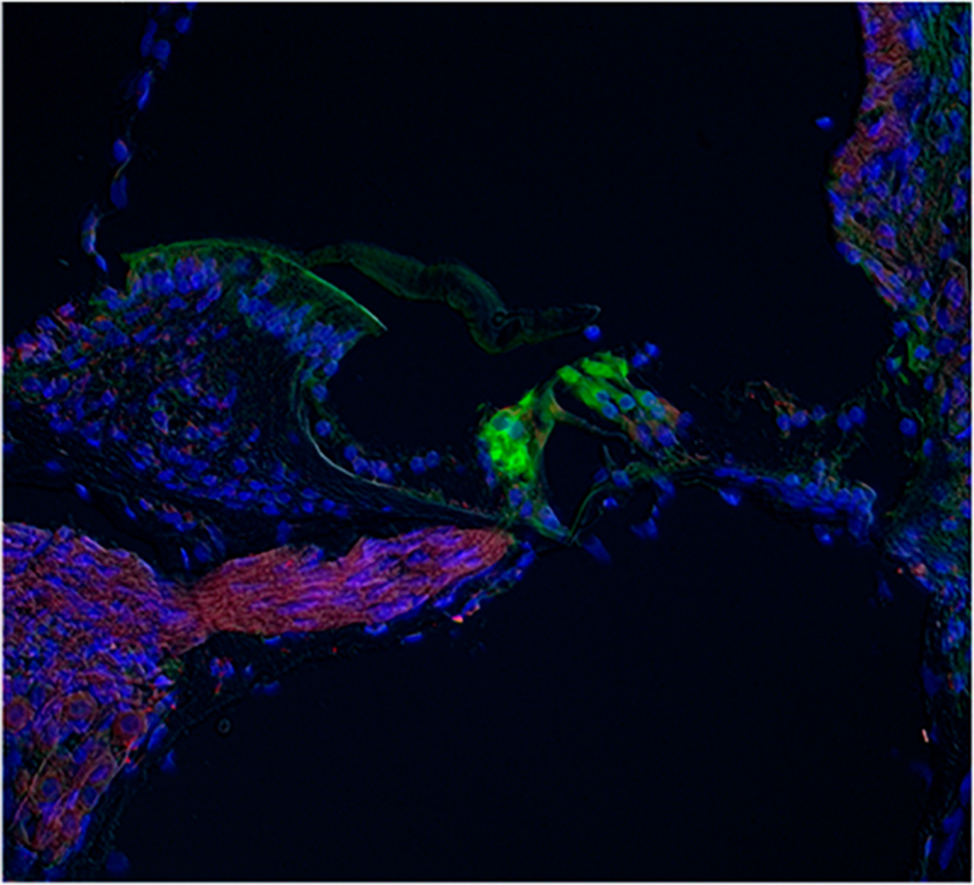
**Immunostaining for ASIC1 in whole cochlea slices.** The IR for ASIC1 (red) was found in the fibers running from spiral ganglion to the OC, and a faint staining at the hair cell base region, most likely due to the presence of afferent terminals. The IR to calmodulin (green) was located at the hair cells. Blue staining corresponds to nuclei stained with DAPI.

Other cochlear structures, such as the tectorial membrane and the stria vascularis, were also stained by the ASIC1, 2 and 4 antibodies. Slices of the whole cochlea showed ASIC1 immunostaining in the fibers running from spiral ganglion to the OC, and a faint staining at the hair cell base region, most likely due to the presence of afferent terminals (**Figure 7**).

### ASIC Activity in Current-Clamp Experiments

**FIGURE 8:**
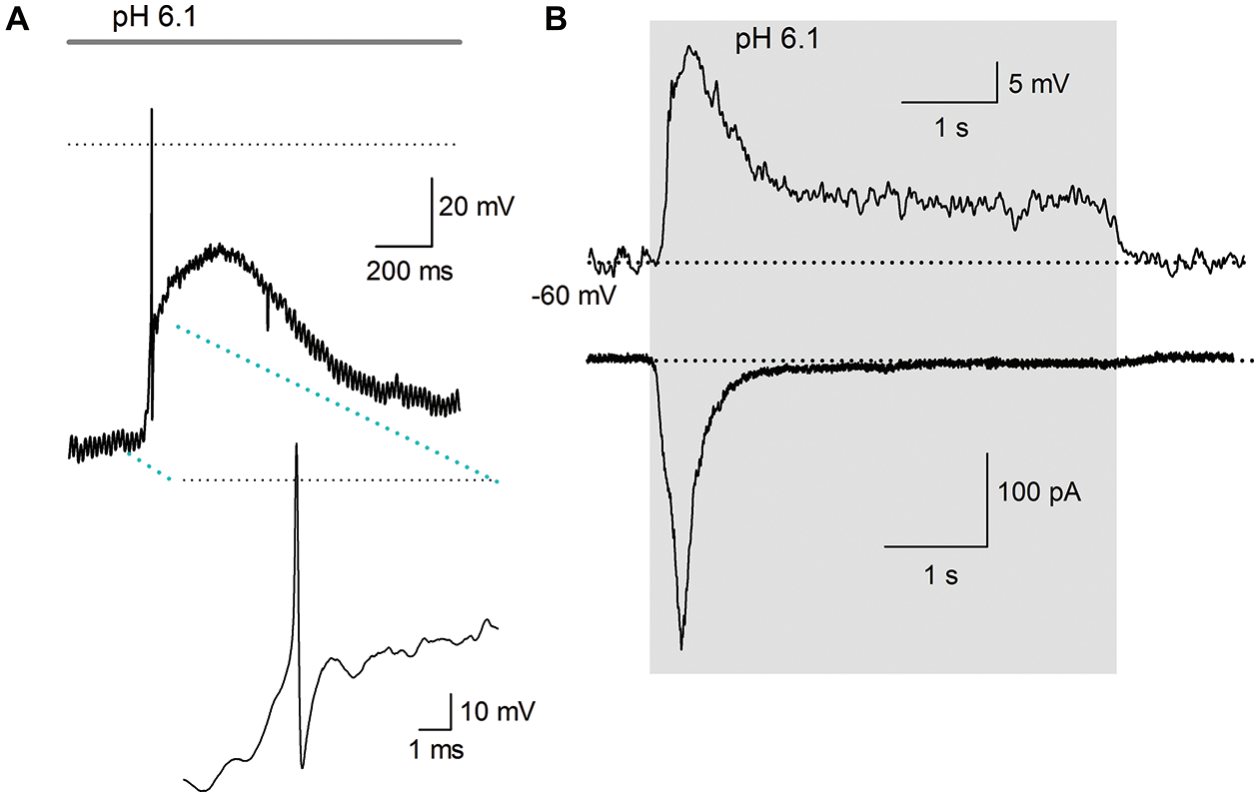
**Action potential discharges are activated by acidic solution. (A)** An AP evoked in response to a pH 6.1 solution; inset shows the AP in larger scale. Only one AP discharge occurred, followed by a large depolarization of about 40 mV. The dotted line represents zero voltage, and line at the top indicates the duration of acidic extracellular perfusion. The membrane potential was set at about -90 mV. **(B)** Current and voltage clamp recording from a cell. Above the current clamp recording shows that acid perfusion (from pH 7.4–6.1 -grey area), evoked no AP discharge although a slowly raising depolarization of >20 mV was induced in response to a pH 6.1 solution perfusion. The time course of the depolarization and its decay neatly follows the time course of the inward current (below) caused by the acidic solution. The dotted line indicates -60 mV for current clamp and 0 pA for voltage clamp recording.

Acidic extracellular solutions of pH 7.0, 6.1, 5.0, 4.5, and 4.0 were used to determine whether APs were generated by extracellular acidification. A total of 40 neurons were recorded, from physiological pH (7.4) and using acidic solutions (pH 6.1 and 4.5) 15% of the neurons fired an AP in response to the acidic pH perfusion (**Figure 8A**); the remaining neurons displayed a sustained pH-dependent depolarization, although the depolarization was above threshold for current discharge (more than 30 mV in some cells), the depolarization rising phase was relatively slow and no AP discharge was induced (**Figure 8B**).

The steady state desensitization curve (**Figure 1A**) indicated that at pH 7.8, there was maximal channel availability and, thus, maximal amplitude of the ASIC current. Therefore, we decided to perform experiments in which cells were bathed in preconditioning extracellular solution of pH 7.8. Under this condition, the pH 6.1 solution induced higher AP firing probability not shown.

**FIGURE 9:**
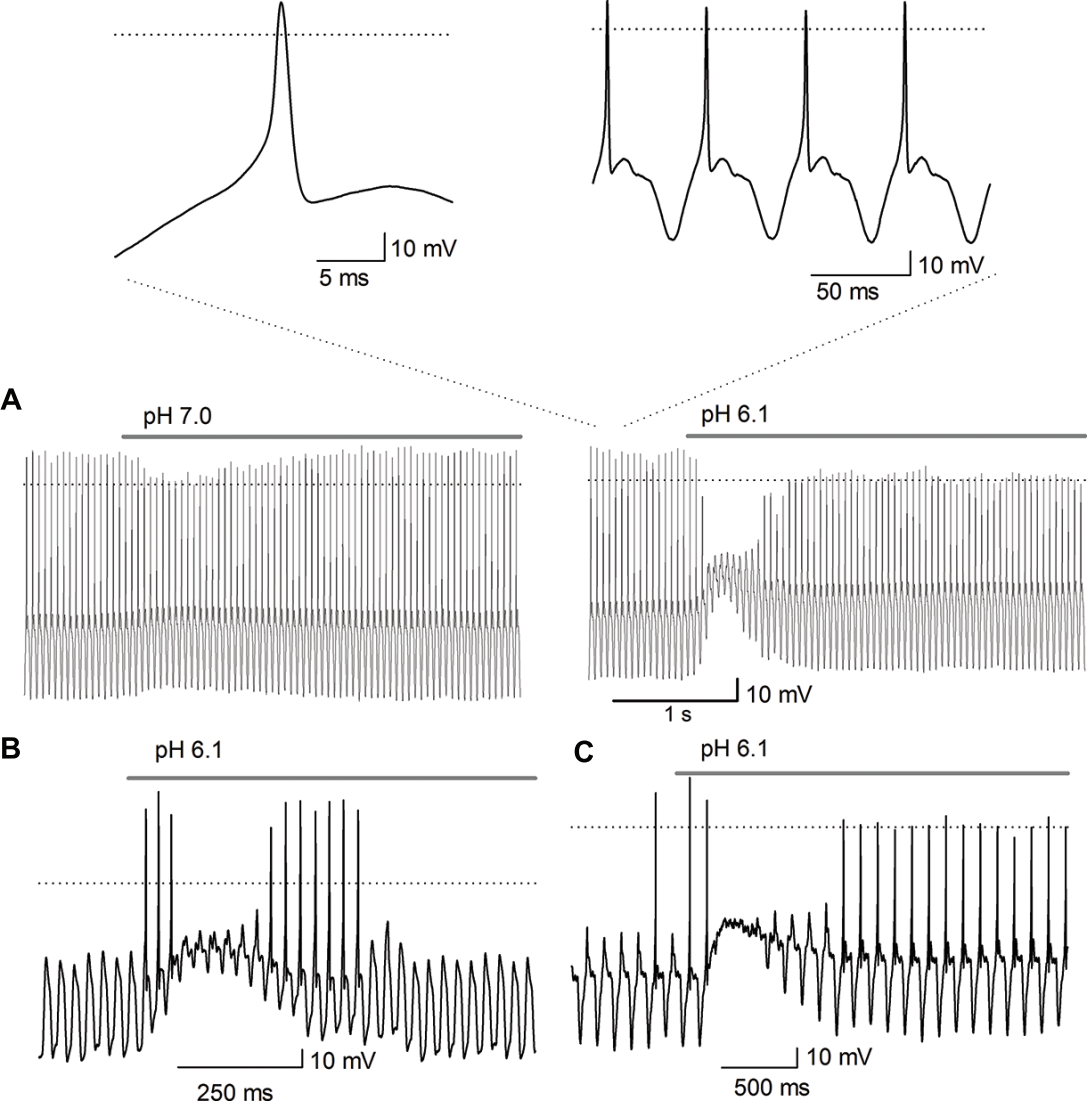
**Effect of acidic pH perfusion on the AP response to sinusoidal stimulation.** The AP discharge was evoked by sinusoidal current injection (20 Hz). **(A)** The depolarization produced by pH 7.0 perfusion reduced the AP amplitude (left trace); pH 6.1 transiently blocked the AP discharge (right trace). The bar above traces indicates perfusion of the acidic solution, either pH 6.1 or pH 7.0. The acidic solution produced a depolarization (about 10 mV for pH 7.0 and of >20 mV for pH 6.1), inhibiting the AP discharge after the beginning of the acidic solution application in a pH-dependent form. The insets above shows the AP in an expanded scale which demonstrate they display a typical AP morphology, with a rapid depolarizing phase, upstroke above 0 mV and repolarization followed by a hyperpolarization period. The AP discharge is phase locked to the sinusoidal stimulation one-cycle to one-AP. **(B,C)** The sinusoidal stimulation was set just below threshold to generate AP. **(B)** The acid perfusion produced a brief burst of AP followed by inhibition of AP discharge during the largest depolarization (accompanied by a significant decrease of input resistance which produced the decay of membrane response to sinusoidal stimuli), and then again a brief burst of APs. **(C)** The acid perfusion produced a brief burst followed by inhibition of AP discharge during the maximal depolarization and followed by sustained discharge during the whole-acid pulse.

To further elucidate the role of ASIC channels in SGN function, square, and sinusoidal currents were injected to induce AP discharges. Square pulses typically produced a single AP discharge and no further spiking, independently of the amplitude of the current used. Response was not modified by acidic solution perfusion although the acidic solutions added a significant depolarization of more than 10 mV. Altering the pH during the injection of sinusoidal current (10–40 Hz) depolarized the cell membrane and decreased the frequency and the amplitude of the APs in those cells stimulated with suprathreshold current injection that generated one AP phase locked in every cycle (*n* = 30). These results revealed a pH dependency of the ASIC current; at a more acidic pH, the depolarization of the SGNs was greater and longer, producing a greater inhibition of the AP discharges (**Figure 9A**). In all those cells stimulated with subthreshold sinusoidal current (*n* = 13) the acid solution perfusion produced APs in the rising phase of the depolarization, that were followed in some cells by no AP discharge during acid pulse perfusion (**Figure 9B**, *n* = 8/13) or by brief depolarization inhibition of the AP followed by a transient or sustained discharge during the whole acid solution perfusion (**Figure 9C**).

### Is the *K*_Na_ Activated by ASIC-mediated Na^+^ Influx?

**FIGURE 10:**
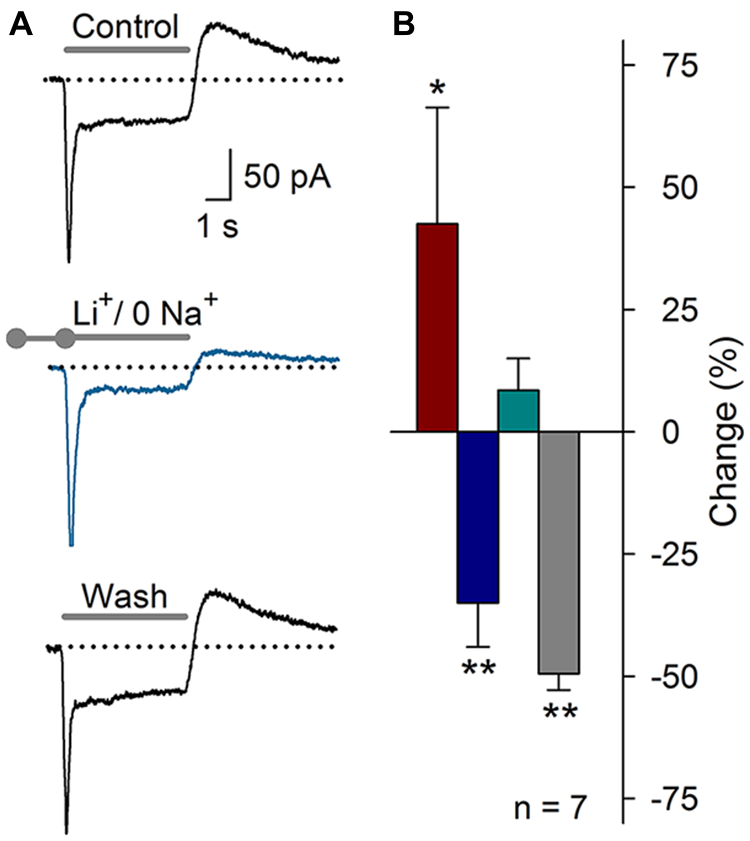
**Substitution of Na^+^ with Li^+^ in the recording solution (pH 7.4 or 6.1). (A)** ASIC current recording under control conditions, after the substitution of extracellular Na^+^ with Li^+^ (blue), or after washout of the Li^+^ solution. The outward current after ASIC activation was clearly reduced. **(B)** Bar graph of the effects of Li^+^ on the ASIC current components (*n* = 7): *I*_peak_ (red), *I*_sus_ (blue), τ_des_ (cyan), and the outward current (grey). The data are presented as the percent-change relative to the control value. (^∗^*P* < 0.05, ^∗∗^*P* < 0.01, and paired Student’s *t*-test).

In 20% of the ASIC current recordings, an outward current component was detected after the acidic pulse. We hypothesized that this outward current may be the *K*_Na_ (Cervantes et al., 2013) that is activated by the Na^+^ influx produced by the ASIC current. To test this hypothesis, extracellular Na^+^ was replaced with Li^+^ (pH 7.4 and 6.1). Sustained Li^+^ perfusion resulted in an increase of the peak of the acid-gated current by 42 ± 23% (*P* < 0.05), a decrease in the *I*_sus_ by 35 ± 9% (*P* < 0.01), and a decrease in the post-ASIC activation outward current by 50 ± 3% (*P* < 0.01, *n* = 7), no change in the τ_des_ was detected (**Figure 10**). This result suggested that, in fact, the Na^+^ influx through ASICs may activate the *K*_Na_ current, thus producing an outward current at the end of the acid-gated current. These results suggest that complex interactions between ASIC-mediated currents and other ionic currents may occur in SGNs.

## Discussion

The H^+^-gated current in mouse SGNs displayed rapid activation, partial desensitization that followed an exponential time course, and a sigmoidal sensitivity to the H^+^ concentration, with a pH_50_ of 6.2. Steady-state desensitization was fitted to a sigmoidal curve with a pH_50_ of 7.3. The activation and steady-state desensitization curves displayed a window current in which a significantly high open probability was detected at pH 6.5–8.0. The proton-gated current was carried by Na^+^; reduced by amiloride, Gd^3+^, low concentrations (nM) of Zn^2+^, and ASA; and enhanced by FMRFamide and high concentrations (μM) of Zn^2+^. We found that all four ASIC mRNAs are expressed in SGNs and the ASIC-1a, -2a, -2b, -3, and -4 proteins are detectable via immunohistochemistry.

Pharmacological analysis revealed that the ASIC1, ASIC2a, and ASIC3 subunits are functionally expressed in SGNs. No drugs acting upon the ASIC1b, 2b, and 4 subunits have been described; thus, no pharmacological evidence for their functional expression was obtained. Regarding the action of Gd^3+^, this ion typically inhibits ASIC3 homomeric and ASIC2a+3 heteromeric channels (Babinski et al., 2000) indicating the functional participation of these two subunits (ASIC2a and 3) in SGNs. FMRFamide peptide has been shown to produce a slowing of the current desensitization and to increase the *I*_sus_ in ASIC channels conformed by ASIC1 and 3 subunits (Askwith et al., 2000). In this study, FMRFamide potentiated the ASIC currents suggesting the functional presence of the ASIC1 and ASIC3 subunits in the SGN. Previous reports have indicated that Zn^2+^ binds to ASIC to a low-affinity site in the extracellular loop of the ASIC2a subunit (His 62 and 339), potentiating the ASIC current (Baron et al., 2001). We found that coapplication of Zn^2+^ in the μM range resulted in an increment of the current amplitude, indicating that ASIC2a is incorporated into SGN ASICs. It has also been shown that sustained perfusion of Zn^2+^ in the μM range inhibits ASIC3 homomeric and heteromeric ASICs (Jiang et al., 2010). In SGNs, sustained application of Zn^2+^ in the μM range increased the τ_des_ but did not affect the *I*_peak_. Zn^2+^ also binds to a high-affinity site on ASIC1a (Lys-133) that may constitutively inhibit the activity of ASIC1a homomers and ASIC1a–2a heteromers (Chu et al., 2004), as most of the salts used to prepare physiological solutions in laboratories contain trace amounts of Zn^2+^ (approximately 20–50 nM; Paoletti et al., 1997). Applying a Zn^2+^ chelator (TPEN) in our experiments produced a significant increase in the ASIC *I*_peak_, indicating the participation of the ASIC1a subunit.

The Zn^2+^-mediated modulation of the ASIC current in SGNs is complex because the ASICs in these neurons are most likely heteromeric. Nanomolar Zn^2+^ concentrations inhibit ASIC1a homomers or heteromers, and micromolar Zn^2+^ concentrations stimulate ASIC2a and ASIC3 homo or heteromers, making it difficult to predict the ultimate effect of Zn^2+^ on these channels. Nevertheless, we identified three possible effects of Zn^2+^, supporting the concept that ASIC1a, 2a, and 3 are functionally expressed in SGNs.

According to our RT-PCR and immunohistochemistry results, all four ASIC subunits are expressed in the SGN. Although no information about their assembly was obtained, these ASICs are most likely assembled as heteromers, which may account for the diversity of the pharmacological effects that we observed. These results coincide with reports showing that when the same cell expresses different ASIC subunits, the assembled ASIC tend to include all of the expressed subunits (Benson et al., 2002; Hesselager et al., 2004). The expression level of ASICs based on RT-PCR was expected because the Asic3 subunit has been shown to be expressed in the PNS (Waldmann et al., 1997b). The higher expression level of Asic3 in the SGNs than in the B is in agreement with a previous study of SGNs (Hildebrand et al., 2004).

The expression and distribution of ASICs based on immunohistochemistry strongly agree with our physiological and pharmacological results, and coincided with previous studies, in which ASIC2a and 3 were detected in SGNs (Hildebrand et al., 2004; Peng et al., 2004). Moreover, electrophysiology and pharmacological evidences demonstrate that ASIC current is still functional in mice after the onset of hearing. Amiloride and FMRFamide effects in P14–15 mice were more potent. These data suggest a change in ASIC subunit expression in SGN after the onset of hearing.

The aminoglycoside antibiotics increased the τ_des_, and the *I*_sus_ of ASIC currents of DRG neurons (Garza et al., 2010) resulting in a higher Na^+^ entry and probably Ca^2+^ entry. These findings were particularly interesting because clinical use of aminoglycoside antibiotics is a major cause of non-genetic hearing loss; mechanisms include hair cell death following intracellular accumulation. These molecules also act directly on ASIC currents in SGN increasing the τ_des_, and the *I*_sus_, this could be a possible mechanism contributing to their ototoxic effect by increasing Na^+^ and Ca^2+^ entry through ASIC channels.

A slowly activating outward after-current was detected following ASIC current activation in 20% of the cells. Activating ASIC channels produces an increase in intracellular Na^+^ concentration, which could activate *K*_Na_ channels to generate an outward after-current. Interestingly, *K*_Na_ channels are not activated by Li^+^ (Bhattacharjee and Kaczmarek, 2005; Cervantes et al., 2013), whereas ASIC channels are highly permeable to this ion (Waldmann et al., 1997a). The use of Li^+^ instead of Na^+^ as an ASIC current carrier significantly decreased the outward after-current, indicating that *K*_Na_ channels activation in SGNs could be coupled to ASIC channels activation.

In current-clamp experiments extracellular acidification induced a significant depolarization of the SGNs and AP discharge in 15% of them. SGN were held at -60 and -90 mV, the most hyperpolarizing voltage was used to increase sodium voltage-gated channel availability. However, no differences were found between these two membrane potentials. Apparently, AP discharge is most likely due to ASIC channels availability, which is less than 40% at pH 7.4 as showed in the desensitization curve (**Figure 1A**). Increasing ASIC channel availability using pH 7.8 extracellular solution resulted in a higher AP firing probability. Thus, ASIC channel availability is playing a role in the SGN excitability, and not only acidification but alkalinization of the media may produce a significant effect in the ASIC current functional role. Failure to evoke AP firing in some cells could be due to the slow rise time of depolarization induced by the ASIC current, giving time to sodium voltage-gated channels to inactivate (Santos-Sacchi, 1993). Also the inhibition of the Na^+^ current may account for the AP inhibition during acid solution perfusion; in fact the decrease of the AP amplitude indicate that this is taken place in our system (Hille, 1971).

In vivo, SGNs basally discharge in response to ongoing synaptic transmission from the cochlear hair cells. To look for modulatory actions of pH on the afferent activity, AP discharge was evoked with sinusoidal current injection and SGN activity modulation by extracellular protons showed that increased acidity depolarized the membrane and reduced the ongoing AP activity. These results are analogous to those in hippocampal neurons, where ASIC activation terminated the AP burst in a pH-dependent manner (Vukicevic and Kellenberger, 2004). However, in most of the cells stimulated with subthreshold sinusoidal current injection, the acid perfusion induced APs discharge during the rising phase of the depolarization and in about 10% of the cells there were a sustained discharge during the whole-acid perfusion (5 s), demonstrating that ASIC activation produces a significant excitatory input to the cochlear afferent neurons.

The ASICs are located in postsynaptic regions (Wemmie et al., 2003, 2008; Ettaiche et al., 2004, 2006). Furthermore, rapid, transient synaptic cleft acidification due to the acid content (pH 5.7) of synaptic vesicles has been observed (Krishtal et al., 1987; Miesenböck et al., 1998; Du et al., 2014). Moreover, in CNS it has been shown that protons and ASIC channels are required for synaptic plasticity (Du et al., 2014; Kreple et al., 2014). Additionally, in the vestibular system a non-quantal excitatory postsynaptic current was caused by cleft acidification (Highstein et al., 2014). Otherwise, there is evidence that protons modulate synaptic transmission and afferent neurons excitability in mammalian vestibular system via ASIC channels (Mercado et al., 2006, 2012; Almanza et al., 2008). In SGN the ASIC channels may be activated by mechanically evoked proton release from hair cells, either via vesicular release of protons along with glutamate (intravesicular pH is 5.7) or by other mechanisms such as Na^+^/H^+^ exchange or electrogenic Na^+^/HCO_3_ cotransport (Diering and Numata, 2014). This is the first report involving all ASIC subunits contributing to the SGN proton gated current covering functional, expression and immunolocalization. The evidence presented shows that protons have a modulatory role in cochlear afferent neurons excitability by acting on ASIC channels and promoting their depolarization.

## Author Contributions

ES and RV designed the project and participated in all aspects of the experimental work. AG-G was a doctoral student and realized the most part of the experiments. FM was postdoctoral researcher who realized part of the PCR and immunohistochemistry experiments. IL contributed with part of the immunohistochemistry experiments. ES, RV, and AG-G written the first draft of the manuscript and all authors contributed to refine and finish the English text of the manuscript.

## Conflict of Interest Statement

The authors declare that the research was conducted in the absence of any commercial or financial relationships that could be construed as a potential conflict of interest.

## Abbreviations

ADI: Alpha Diagnostic International
AP: action potential
ASA: acetylsalicylic acid
ASIC: acid-sensing ion channel
B: brain
BSA: bovine serum albumin
cDNA: complementary deoxyribonucleic acid
CNS: central nervous system
DAB: diaminobenzidine
DNA: deoxyribonucleic acid
DRG: dorsal root ganglia
ENaC/DEG: epithelial sodium channels/degenerin
HEPES: 4-(2-hydroxyethyl)-1-piperazineethanesulfonic acid
IHC: inner hair cell
*I*_int_: integral of the current
*I*_peak_: peak current
IR: immunoreactivity
*I*_sus_: sustained current
*K*_Na_: Na^+^-activated potassium current
MES: 2-(*N*-morpholino) ethanesulfonic acid
Neo: neomycin
OC: organ of Corti
PBS: phosphate buffer solution
PNS: peripheral nervous system
RNA: ribonucleic acid
mRNA: messenger ribonucleic acid
SG: spiral ganglion
SGN: spiral ganglion neuron
St: streptomycin
TPEN: *N*,*N*,*N*’,*N*’–tetrakis-(2-piridilmetil)-etilendiamina
*τ*_des_: desensitization tau
